# Nanocarrier system: An emerging strategy for bioactive peptide delivery

**DOI:** 10.3389/fnut.2022.1050647

**Published:** 2022-12-05

**Authors:** Xu Zhang, Xinshe Li, Yunhao Zhao, Qing Zheng, Qiang Wu, Yougui Yu

**Affiliations:** College of Food and Chemical Engineering, Shaoyang University, Shaoyang, China

**Keywords:** bioactive peptide, delivery system, nanoparticle, peptide drug, bioavailability

## Abstract

Compared with small-molecule synthetic drugs, bioactive peptides have desirable advantages in efficiency, selectivity, safety, tolerance, and side effects, which are accepted by attracting extensive attention from researchers in food, medicine, and other fields. However, unacceptable barriers, including mucus barrier, digestive enzyme barrier, and epithelial barrier, cause the weakening or the loss of bioavailability and biostability of bioactive peptides. The nanocarrier system for bioactive peptide delivery needs to be further probed. We provide a comprehensive update on the application of versatile delivery systems for embedding bioactive peptides, including liposomes, polymer nanoparticles, polysaccharides, hydrogels, and self-emulsifying delivery systems, and further clarify their structural characterization, advantages, and disadvantages as delivery systems. It aims to provide a reference for the maximum utilization of bioactive peptides. It is expected to be an effective strategy for improving the bioavailability and biostability of bioactive peptides.

## Introduction

Recently, researchers have focused on bioactive peptides from various origins, including animal (1), plant (2, 3), marine organisms (4, 5), bean products (6), milk (7), and fermented products (8). They are generally composed of 2–50 amino acid residues in different combinations and arrangements forming linear or cyclic structures (9). In addition, bioactive peptides have a variety of human body metabolism and physiological regulation functions, which are easy to digest and absorb, and have the effects of promoting immunity, hormone regulation, antibacterial, antiviral, decreasing blood pressure, and reducing blood lipid (9–11). They are widely used in food, medicine, and other fields. Particularly, it is the most popular research topic and functional factor with great development prospects in the current international food industry.

**Figure 1:**
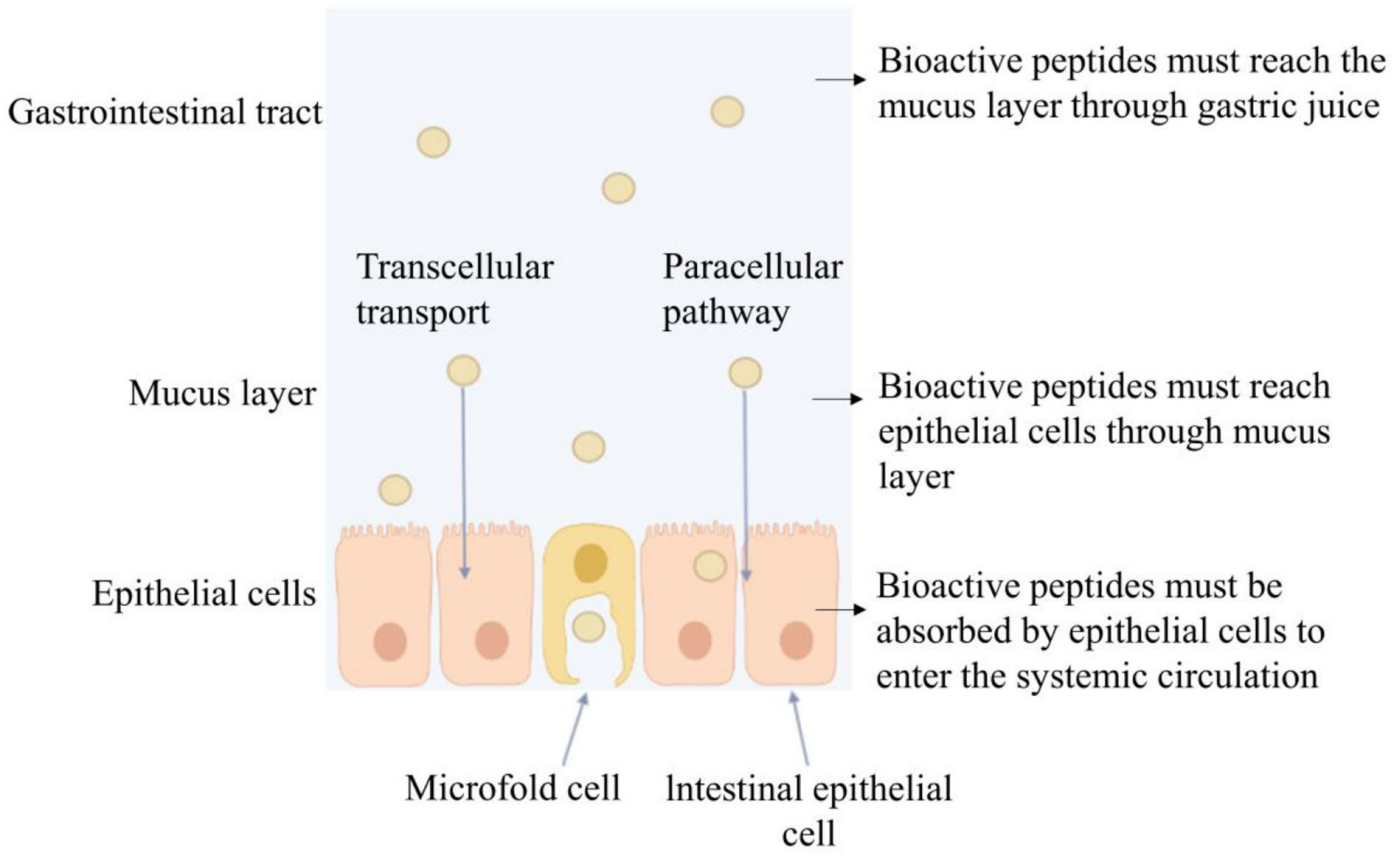
Three barriers to bioactive peptide absorption.

Bioactive peptides play an important role in cancer treatment, new drug screening, vaccine development, and nutritional drug delivery and are considered desired substitutes for drugs. Compared with macromolecular proteins, bioactive peptides have the advantages of high activity, tissue permeability, and weak immunogenicity under the same unit mass conditions. Compared with small molecular substances, bioactive peptides have stronger potency, selectivity, specificity, and lower cytotoxicity and drug interactions (12). Oral administration is a direct and effective way of bioactive peptide administration. However, after oral administration, bioactive peptides may be degraded by gastric acidic secretion, digestive enzymes, intestinal brush border membrane, and intestinal epithelial cell endopeptidase in the gastrointestinal tract (13). During oral administration, it is possible that bioactive peptides are hydrolyzed by 40 kinds of enzymes at least, causing subsequent loss of bioactivity before reaching systemic circulation. It was reported that most food protein-derived bioactive peptides containing more than two–three amino acid residues do not withstand simulated gastrointestinal enzymatic digestion (14). Although dipeptides and tripeptides can be fully absorbed through the synergistic transport mechanism of peptide transporters (PepT1 and PepT2), and the brush border membrane Na^+^/ H^+^ exchange transport system, or enter cells through endocytosis and cell bypass (15) as shown in Figure 1, it is still degraded into free amino acids by peptidases in blood and lose bioactivity (16). We studied the gastrointestinal digestion stability of bioactive peptides and found that the peptide composed of amino acid residues such as Phe, Tyr, Val, Leu, Arg, and Lys were not tolerant to gastrointestinal fluid digestion (5, 17, 18). It was reported that after intravenous injection of angiotensin-converting enzyme (ACE) inhibitory peptide Ile-Val-Tyr in the spontaneously hypertensive rat (SHR), it can be degraded into dipeptide Val-Tyr by the aminopeptidase existing in the blood. After intravenous injection, Ile-Val-Tyr had a better blood pressure reduction effect and could maintain for 15 min. Val-Tyr alone had an acute hypotension effect, but the blood pressure returned to normal after 5 min of injection (19). Therefore, the bioavailability of bioactive peptides depends directly on their amino acid sequence. When entering the body to perform an anti-hypertensive effect, it is likely to be degraded and weakened or lost activity.

The undesirable bioavailability of bioactive peptides has prompted researchers to explore efficient methods for complete delivery. Encapsulation of bioactive peptides in nanoparticles is an effective way to prevent their degradation and enhance their bioactivity (20). Many nanocarrier systems have been developed, including lipid particles, polysaccharide particles, biopolymer nanoparticles, and colloidal particles, each of which has its own advantages and limitations (21). There are various administration ways including subcutaneous injection, intravenous injection, and oral administration. In consideration of bioactive peptides derived from food resources, in general, used as functional foods and nutritional supplements, oral administration is preferential (22). Therefore, this study reviewed oral delivery systems of bioactive peptides. Furthermore, the structural characterization and application status of these delivery systems were summarized, and their advantages and disadvantages were analyzed, in order to provide a reference for the application of bioactive peptides.

## Liposome carrier applied in bioactive peptide delivery

### Structural characterization of the liposome delivery system

**Figure 2:**
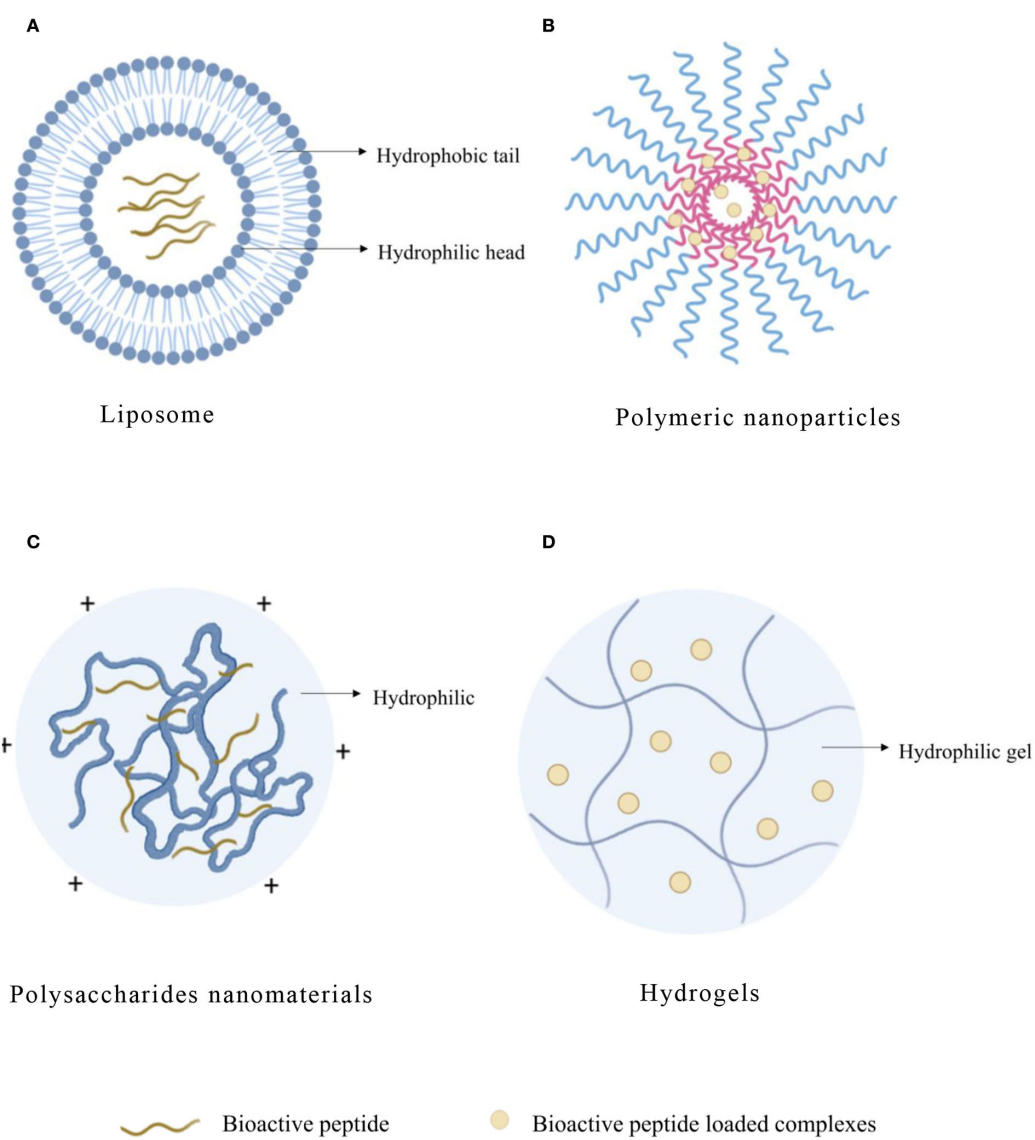
Typical structure of bioactive peptide carriers. **(A)** Liposome. **(B)** Polymeric nanoparticles. **(C)** Polysaccharides nanomaterials. **(D)** Hydrogels.

Liposome, as an efficient organic carrier, is a kind of closed vesicle similar to a biofilm structure formed by the inclusion of phospholipid and cholesterol. When the liposome is dispersed in the aqueous phase, the hydrophobic groups will spontaneously aggregate together under the hydrophobic interaction, and the hydrophilic groups will also aggregate with each other. When the system is stable, the closed ring multilayer structure of “head to head, tail to tail” will be formed (Figure 2A). The interactions between peptide and liposome depend on the hydrophobicity of the peptide, and the structure of the interfacial head-group region of the lipid bilayers of liposome was also important (23). Phospholipid-forming liposome is eliminated slowly in the blood, so drugs loaded on liposome remain in the blood circulation system for a long time. In addition, liposomes can dissolve in the cell membrane of the target organs or tissue to fulfill intracellular transportation of drugs and, thus, improve the bioavailability and stability of drugs. Liposomes contain polar, nonpolar, and amphiphilic regions in the same colloidal particles, so they are particularly suitable for the encapsulation of active peptides (24). The liposome has an aqueous solution core surrounded by a hydrophobic membrane in the form of a lipid bilayer. Hydrophilic solutes dissolved in the nucleus are not easily associated with the bilayer by hydrophobic chemicals. Therefore, liposomes can load hydrophobic and/or hydrophilic molecules. Moreover, amphiphilic peptides locate on the interface between the shell and core of the liposome structure, which would interact with the hydrophobic and hydrophilic amino acid residues, respectively. The liposome is similar to cell membranes and is favorable for the delivery of bioactive compounds, which can otherwise be degraded by the physiological environment (25).

### Practical application of the liposome delivery system

The liposome delivery system has been used to package, protect, and deliver bioactive peptides for the prevention or treatment of diseases in the pharmaceutical industry (26). KRX29 is a small cyclic peptide with potential pathological effects in the treatment of heart failure, which is an effective and selective G protein-coupled receptor kinase 2 (GRK2) activity inhibitor. Although KRX29 presents a small molecular weight (eight amino acids) and can be performed in oral and intravenous administration, it has certain disadvantages including low absorption, unstable physiological environment, and short half-life. It needs to be wrapped in suitable carriers to overcome these limitations. It was found that the bioavailability and stability of drugs could be improved by wrapping into the nano-sized liposomes by thin film hydration and then by ultrasonic-assisted size reduction process (27). In addition, the encapsulated Arg-Leu-Ser-Phe-Asn-Pro (RLSFNP), a hexapeptide milk-derived angiotensin-converting enzyme (ACE) inhibitory peptide, into liposomes with remarkable sustained release and storage capacity can be directly absorbed by Caco-2 cells (28). It has also been found that muramyl peptides encapsulated in liposomes can kill macrophages more effectively than unencapsulated ones (29). Rezaei et al. found that the mutant N-terminal peptide with a zinc binding ring replaced by a disulfide bond ring (ES-SS peptide) has anti-angiogenesis and antitumor properties compared with natural peptides. In order to improve the stability and plasma half-life of ES-SS peptide, ES-SS peptide nanoliposomes with different phosphatidylcholine (PC) ratios were synthesized. ES-SS peptide was gradually released by liposomes. Compared with free peptides, liposome peptide preparation has a higher cell survival rate (30). Heuts et al. (31) studied the possibility of carrying synthetic long peptide (SLP) antigen on liposomes based on 1,2-dioleoyl-3-trimethylammonium propane (DOTAP) and further verified the potential of DOTAP liposomes as a vaccine delivery platform for therapeutic cancer vaccines. Another study also found that P5HER-2/neu derived peptide (P5) in DOTAP-cholesterol drug liposomes injected with CpG-ODN significantly enhanced the response of cytotoxic T cells and highly inhibited the progression of tumors (32). The major problems limiting the widespread use of liposomes are stability, encapsulation efficiency, and lysosomal degradation. Chemical instability may be caused by the hydrolysis of ester bonds and/or oxidation of unsaturated acyl chains of lipids. Liposomal drug formulation could only be affirmative if the encapsulation efficiency is such that therapeutic doses could be delivered in a reasonable amount of lipid, since lipids in high doses may be toxic and also cause non-linear (saturable) pharmacokinetics. Liposomes may deliver the drug to the cells selectively, but the pharmacological activity depends on the ability of the intact drug against degradation to diffuse into the cytoplasm from the endosomes in sufficient amounts (33, 34).

Micelles are unilamellar vesicles made up of amphiphilic polymeric molecules with a hydrophobic core hidden inside the structure and a hydrophilic shell directed outwards with a size range of 10–100 nm (35). Drugs are loaded into micelles either through chemical covalent bonding or through physical encapsulation. Micelles have many advantages as drug delivery carriers, including ease of preparation, stability under physiological conditions, prolonged shelf life, extended circulation time in blood and biological fluids, increased bioavailability, and enhanced pharmacokinetics and biodistribution properties (36, 37). The peptide amphiphile micelles are small spherical particles able to stably associate with cells, making them attractive for the targeted delivery *via* the vasculature, and induce significant peptide-based toxicity in human lymphoma cells (38). Peptide amphiphile micelles (PAMs) are attractive vehicles for the delivery of a variety of therapeutic and prophylactic peptides (39). Furthermore, one cleavage-inducing target of interest could be glutathione, the primary antioxidant in the body, which is present in low concentrations in the blood, but much higher concentrations within cells (40–42). In the food industry, liposomal delivery of proteins, enzymes, vitamins, antioxidants, and seasonings has been studied (43). It was reported that encapsulation of ghrelin (a bioactive polypeptide) into liposomes can improve its chemical stability and increase the retention time of this appetite-suppressing hormone in the blood (44). Liposomes are also used to encapsulate bioactive peptides extracted from hydrolyzed fish protein (45). Liposome encapsulation has been proven to be an appropriate method to reduce the bitterness of bioactive peptides (46). Montero et al. wrapped the shrimp polypeptide component (ST1) with biological activity, including ABTS free radical scavenging ability, angiotensin-converting enzyme activity, and dipeptidyl polypeptidase-IV inhibitory activity, into partially purified soybean phospholipid nanoliposome (L-ST1). The soybean-derived phospholipid-forming liposomes can be bound to the edible membrane of sodium caseinate without significantly losing the integrity of the vesicle structure, thereby increasing the water solubility of the liposome delivery system. Furthermore, the increase in membrane humidity and the presence of liposomes are beneficial to the adhesion of the membrane, especially when they are loaded with polypeptides (47). It has shown that the inclusion of bacteriocins in liposomes can control food spoilage and pathogens and increase the effective safety and shelf life of some foods, as antimicrobial agents can coexist in the aqueous and lipid phases of liposomes (48). The large-scale production of liposome carry is a distinct advantage compared with other carrier systems. Liposomes adapted from the pharmaceutical industry have certain shortcomings in functional food applications. Particularly, the thermal instability of liposome-encapsulated food-derived peptides beyond the phase transition temperature of the phospholipid can limit their incorporation in thermally processed food (49).

The characteristics for applications of liposomes are shown in Table 1. Compared with other embedding techniques, the dominant advantage of the liposome delivery system is that they endow the substances with water-soluble and stability in high water activity (50). Liposomes can also protect the drugs against degradation and integrally release them into target organs or tissues that need to be treated so that the drug concentration in these target organs or tissues is increased, and the therapeutic effect of drugs can be improved. In addition, a drug-loaded liposome delivery system presents resistance to metabolism and degradation by intracellular enzymes, less adverse reaction, and benefits on biocompatibility, targeting ability, and controlled release of the drug. Liposomes have been shown to be stable in embedding materials free from a range of environmental and chemical changes, including enzyme and chemical modifications, as well as buffers against extreme pH and temperature (51). The phospholipids making up liposomes can be commonly found in nature, such as in soybean and egg yolks. However, these are difficult to use in clinical settings due to stability and contamination risks (52).

**Table 1:** Application and characterization of liposomes.

**Usage**	**Trade name**	**Liposome platform**	**Advantage**	**Disadvantage**	**References**
Antibacterial drug carrier	Ambisome^®^	HSPC: Cholesterol: DSPG (2:1:0.8)	1. Good targeting ability	1. Higher cost of industrial production	(23)
	Fungisome^®^	PC: Cholesterol (7:3)	2. Good biocompatibility 3. Synergistic and attenuated effects 4. Delay or control the release of drugs in the tissues 5. Less adverse reactions	2. Low stability 3. Low encapsulation efficiency for some water-soluble drugs 4. The targeting of general liposomes is mainly concentrated in organs rich in reticular endothelial cells such as liver, kidney and spleen. If other tissues and organs are treated, the targeting is not obvious	(24) (25) (123) (124) (125)
Anti-tumor drug carrier	Onivyde^®^	DSPC:Cholesterol:MPEG- 2000-DSPE (3:2:0.015)			
	Doxil^®^	HSPC:Cholesterol:PEG 2000-DSPE (56:38:5)			
Anti-cardiovascular and cerebrovascular diseases drugs carrier	SSL-PAESe	DSPC:Chol:DSPE-PEG (9:5:1)			
Analgesic	DepoDur™	DepoFoam™			
	Exparel^®^	DepoFoam™			
Vaccine adjuvant	Inflexal^®^	Unknow			
Antidote	metHb@Lipo	Unknow			

## Solid lipid nanoparticles applied in bioactive peptide delivery

### Structural characterization of solid lipid nanoparticle

Solid lipid nanoparticle (SLN) is made of natural, semi-synthetic, or synthetic lipids, which contain triglycerides, fatty acids, phospholipids, and steroids, and is widely considered a safe and biodegradable carrier (53). SLNS is a promising technology for the targeted delivery of anticancer drugs (54). The main disadvantage of SLNS is that the encapsulation efficiency is affected by the polymorph structure of solid lipids. If SLN is composed of high-purity single lipids, a perfect lattice may be formed during storage, which may reduce the solubility of drugs in the lipid matrix and eventually lead to the expulsion of encapsulated drugs. When SLN forms, lipids crystallize in disordered α and β crystal structures. During storage, lipids are gradually arranged in a more stable and orderly manner, resulting in β′ and β crystalline forms from which encapsulated drugs can be discharged (55). Drug-loaded SLN combines the best characteristics of polymer nanocarrier and liposome delivery system. It maximizes drug targeting and creates uniform particle distribution, thus providing low toxicity and good histocompatibility, but it is easy to be degraded. Therefore, the utilization efficiency is poor, and it cannot guarantee that the embedded SLN can reach the target organ and tissue and then release it (Table 2).

**Table 2:** Application and characterization of solid lipid nanoparticles.

**Solid lipid microspheres**	**Efficiency**	**Advantage**	**Disadvantage**	**References**
Lidocaine in beeswax-chitosan microparticles	LC: 5% EE: 49%	1.Better biocompatibility 2. Physical stability 3. Low toxicity 4. Green synthesis (no organic solvents are required in their preparation)	1. Easily degraded, low utilization efficiency 2. It is not completely guaranteed that the embedded material can reach the effective site of action and then be released 3. Poor stability 4. Low incorporation capacity	(53) (54) (55) (126) (127) (54)
Ibuprofen in beeswax-starch microparticles	LC: 20% EE: 90%			
Technique of HIP formation and O/O emulsion-evaporation	EE: 74.6%			
Leuprolide-docusate HIP were encapsulated in SLN	EE: 75%.			

EE, entrapment efficiency; LC, loading capacity.

### Practical application of solid lipid nanoparticle

Dumont et al. found that SLN is a promising system to overcome the inherent barriers of stability and bioavailability of the peptide by oral administration. It has been proved that SLN can encapsulate peptides after lipidation without peptide denaturation (56). Christophersen et al. (57) studied the degradation mechanisms of peptide-loaded SLNs containing different types of lipid excipients and found that the release rate became faster after lysozyme was added. However, the hydrophobicity of SLN limits the encapsulation of hydrophilic peptides. Su et al. (58) found that SLN could effectively maintain the stability of bioactive peptides in gastrointestinal digestion. In addition, the macromolecular peptide could be successfully wrapped in SLN and nanocrystallized, thereby improving their physical and chemical stability. In particular, the encapsulation in SLN protects the secondary hydrolysis of bioactive peptides in simulated gastrointestinal fluid, which helps maintain their dipeptidyl peptidase IV (DPP4) inhibitory function. It has encapsulated water-soluble peptides in biodegradable and biocompatible SLN, but the encapsulated peptides are easily released in simulated gastrointestinal fluid in the presence of proteases (59). SLN is a potential carrier system for transporting protein-type antigen vaccines while avoiding the use of organic solvents (60). Kadari et al. (61) found that angiopep-2-modified SLN was an excellently targeted drug delivery system for glioma therapy. SLN could be used to transport antioxidant glutathione (GSH) to immunocompetent fish cells, and the antioxidant activity of GSH *in vitro* is stable in the transport process (62).

Solid lipid nanoparticle-engineered nanostructured lipid carrier is formulated. Insulin and insulin analog—glargine insulin—are entrapped in the lipid matrix through hydrophobic ion pairing. Studies *in vitro* and *in vivo* were carried out by employing fluorescently labeled peptides. The superiority of glargine insulin-loaded nanostructured lipid carrier demonstrated significantly higher permeation (till 30% dose/ml) compared to the free peptide (63). Xu et al. (64) developed functional nanocarriers for efficient oral administration of biological macromolecules, and the developed SLN was proved to increase serum insulin concentration and produced good hypoglycemic reaction. In addition, the prepared insulin-loaded cationic solid lipid nanoparticle (CSLN) by water-in-oil emulsion technology can protect the encapsulated insulin from the degradation of pepsin and trypsin and improve its biostability (65). Hydrophobic ion pairing of leuprolide and insulin, chosen as model peptides, allowed the encapsulation of these molecules in SLN produced through the coacervation technique, nanoparticles *in vitro* can be used as a polypeptide sustained release system, maintaining the integrity of the peptide (66). SLN prepared by thermo-high pressure homogenization method has the advantages of good physical stability, protection of mixed unstable active substances, and controlled release, which is very suitable as a carrier for transporting active components to the skin. Furthermore, SLN technology allows polypeptides to enter the skin to play a protective role (67).

Since the basic template of SLNs is a solid lipid, lipophilic drugs have more affinity toward SLNs than hydrophilic ones. For the preparation of SLNs with lipophilic drugs, oil-in-water (O/W) emulsions are preferred. For the encapsulation of hydrophilic drugs, multiple emulsion systems such as water/oil/water (W/O/W) emulsion with or without the use of organic solvents are reported (68). Furthermore, water-in-oil high internal phase emulsions (W/O HIPEs) could be successfully used to encapsulate bitter peptides, which reduced their bitterness and improved their gastric stability (69). Double emulsion of the water-in-oil-in-water (W_1_/O/W_2_) type is one of the carrier systems used for encapsulation, protection, and delivery of both hydrophilic and hydrophobic active components (70). Recently, double emulsions have been studied in the delivery of hydrophilic bioactive compounds in the food and pharmaceutical industries (71, 72). Among the different bioactive peptide concentrations (1%−20%), 5% of bioactive peptides was determined to be the amount to encapsulate in the W_1_ phase in the double emulsion that would ensure emulsion droplet stability (72).

## The self-emulsifying carrier applied in bioactive peptide delivery

### Structural characterization of self-emulsifying drug delivery system

Self-emulsifying drug delivery system (SEDDS) is a lipid carrier, which is spontaneously formed by the isotropic mixture of oil, surfactant, and cosurfactant through mixing with water (73). SEDDS has high biocompatibility, which can protect polypeptides from degradation and increase membrane permeability. It has been widely used to improve the oral bioavailability of insoluble drugs, which can help overcome the obstacles encountered in the oral administration of hydrophilic macromolecules, namely, intestinal protease degradation, poor mucus permeability, and low cellular uptake (74). It has been proved that pepsin, trypsin, chymotrypsin, elastase, and other hydrolases are not soluble in oily SEDDS droplets. Therefore, due to the hydrophilicity of peptidases and proteolytic enzymes, they cannot enter the lipid droplets of SEDDS containing bioactive peptides, which can protect them against enzymatic degradation (75, 76). Compared with other nanocarriers, SEDDS preparation is simpler and more cost-effective. At present, the self-emulsifying method has been successfully applied to the transportation of anticancer drugs, antiviral drugs, antibacterial drugs, immunosuppressants, and natural products such as antioxidants and alkaloids (Table 3).

**Table 3:** Application and characterization of self-emulsifying drug delivery systems.

**Delivery system**	**Bioavailability**	**Advantage**	**Disadvantage**	**Application**	**References**
SNEDDS for noninvasive protein delivery	Increased by 1.29-fold compared to BLM free solution	1. Higher mucus penetration capacity 2. Industrial scale scaling and ease of manufacture 3. More cost-effective	1. Instances of lack of correlation between the in vitro and in vivo results 2. Lack of robust in vitro model 3. High production cost 4. Low portability, 5. Low drug loading 6. Physico-chemical instability of drug and vehicle components	Delivery of anti-cancer agents, anti-viral drugs, anti-bacterial, immunosuppressant, and natural products such as antioxidants and alkaloids	(73) (74) (75) (128) (129) (130)
Self-emulsifying hybrid microparticles	Increased by 154%				
Octreotide-deoxycholate SEDDS formulation	Close to 5%				
Exenatide-n-octadecyl sulfate into SEDDS	19.6%				
Docusate HIP into SEDDS	15.2%				
The SEDDS containing octreotide/sodium deoxycholate HIP	5%				

### Practical application of self-emulsifying drug delivery system

Self-emulsifying drug delivery system is discovered for the oral administration of hydrophilic drugs such as peptides, proteins, polysaccharides, and pDNA (77). Due to hydrophobic ion pairing (HIP) with oppositely charged lipophilic auxiliary agents, the resulting complexes can be incorporated into the lipophilic phase of SEDDS. Depending on the solubility of the complex in the pre-concentrate SEDDS, drug release can be adjusted on purpose by choosing more or less lipophilic auxiliary agents in appropriate quantities for the HIP. Within the oily droplets formed in the gastrointestinal tract, drugs are protected from degradation by proteases and nucleases and thiol-disulfide exchange reactions with dietary proteins. The oily droplets can be made mucoadhesive or highly mucus permeating depending on their target site. Furthermore, it can be tuned by adjusting their zeta potential or decorating them with cell-penetrating peptides (74).

Sustained release of bioactive peptides from SEDDS seems essential to avoid their degradation in the gastrointestinal tract. SEDDS has been shown to penetrate effectively into the intestinal mucus gel layer, making peptides or protein drugs closely in contact with the absorption membrane (73). Compared with other drug delivery systems, SEDDS is the most effective mucus permeation system. *In vitro* studies showed that the penetration of exenatide containing SEDDS was 2.7 times higher than that of drugs without SEDDS. *In vivo* evaluation experiment showed that compared with the control group, the area under plasma concentration curves (AUC) of blood glucose in the SEDDS group was decreased by 20.6% (78). Zupančič et al. (79) found that SEDDS showed mucus permeability and had protective effects on the degradation of trypsin, α-chymotrypsin, elastase, simulated gastric juice, and simulated intestinal juice. Mahmood et al. added hydrophilic macromolecular drugs into SEDDS, which could protect them from the enzyme and thiol barriers in the gastrointestinal tract. In addition, SEDDS can penetrate the mucus gel barrier in a relatively effective way to promote the transport of the combined hydrophilic macromolecular drugs to the underlying epithelial cells (74).

## Polymeric nanoparticles applied in bioactive peptide delivery

### Structural characterization of polymeric nanoparticle

Polymeric nanoparticle (NP) refers to a solid particle with a size range from 10 to 1,000 nm, which can encapsulate drugs in the polymer matrix to prevent them from being degraded by enzymes (Figure 2B). Compared with liposome and SLN, NP also showed higher stability in biological fluids (Table 4). In addition, it is a promising delivery system for proteins and peptides because of the versatility, sustained release, protection of encapsulated proteins, and peptides from enzymatic degradation and biocompatibility of nanoparticles (80). Polymers have great functionalization ability and can be used to couple targeted ligands to improve biological distribution (81). NP can also compensate for the disadvantages of peptide treatment by increasing bioavailability, exhibiting controlled and sustained release, and enhancing cellular uptake (82). In addition, compared with other particles, NPs can effectively transfer across the epithelial surface (83). The polymer matrix can protect peptides from degradation and provide a desirable controlled release of peptides (84).

**Table 4:** Application and characterization of polymeric nanoparticles.

**Delivery system**	**Bioavailability**	**Advantage**	**Disadvantage**	**References**
PEG-coated lipid-based nanocarriers	Increased to 120 and 182%	1. Good pharmacokinetics and drug release characteristics 2. Higher stability	1. Poor biocompatibility 2. High production cost	(80) (81) (82)
Nanoparticles made of a mixture of albumin and dextran and cross-linked using sodium trimetaphosphate	77%			(83) (84) (131) (132)
Labrasol^®^	7% in the jejunum and 12% in the colon.			
RaniPill™, in a size 000 hydroxypropyl methylcellulose capsule	65 ± 9% (without the impact of food on its performance)			
Melanotan II (MTII) was incorporated into thermally hydrocarbonized nanoporous silicon (THCPSi)	15%			

Polylactic-glycolic acid copolymer (PLGA) is a very important synthetic polymer material for preparing polymeric nanoparticles, which can achieve safe and effective delivery of drugs without causing tissue damage (85, 86). It has been confirmed that PLGA-based nanoparticles (PLGANPs) have a smaller particle size, better environmental adaptability, and biocompatibility than hydrogels, alginates, and chitosan (CS) polymer-coated particles (87). In addition, the adhesion and cell uptake of PLGANPs can be significantly improved by functionalizing positively charged polymers or proteins on the surface of particles (88–90).

Rastogi et al. used a modified s/o/w method to prepare insulin-loaded polycaprolactone-polymer poly (ethylene glycol)-polycaprolactone (PCL-PEG-PCL) nanoparticles in order to obtain higher drug loading. Insulin was hydrophobically modified by sodium deoxycholate. After pairing with sodium deoxycholate, the lipophilicity of insulin was increased by five times, and the encapsulation efficiency was increased by 10%−50% (91). In addition, polypeptide encapsulated with nanoparticles based on polymer poly (ethylene glycol)-block-polycaprolactone (PEG-b-PCL) presented the cytotoxicity induced by polyglutamine (80). Furthermore, Kelly et al. (92) screened and developed porous silicon nanoparticle (PSNP) composites for micRNA inhibition of intracellular delivery of peptide nucleic acid (PNA).

### Practical application of polymeric nanoparticle

A recent study has shown that polyethylene glycol (PEG) embedding can be used as a protective method or a ligand competition for antimicrobial peptide BP100 transposition (93). It is suggested the feasibility of applying antimicrobial peptides carried on PEG-capped NPs for possible treatments of lung diseases. The anticancer peptide NuBCP-9 was encapsulated in NPs to enhance efficacy and avoid drug resistance, which was enhanced by increasing the PEG chain length in the block copolymers (94). Hoshino et al. confirmed that NPs accelerated the clearance of toxic peptides, which is a 26 amino acid cytolytic peptide isolated from bee venom, and finally accumulated in liver macrophages. PEG hydrophilic polymer particles also help to improve the biocompatibility of synthetic NPs and enhance their stability in plasma, revealing the potential and possible limitations of using synthetic ones as a plastic antidote (95). Cellulose nanocrystal (CNC) is a natural, non-toxic, and neutral material with a rod-like or spherical construction. It has been applied in various fields such as pharmaceuticals, food industry, paper industry, composites, and polymer composites (96). Cellulose-based composite macrogels made by cellulose fiber/cellulose nanofiber (CCNM) are used as an intestine delivery vehicle for probiotics (97).

## Polysaccharide nanomaterial applied in bioactive peptide delivery

### Structural characterization of polysaccharides nanomaterial

Polymer-based nanocarriers can be divided into natural polymers and synthetic polymers. Chitosan (CS) is a kind of natural polysaccharide isolated from the excrement of crustaceans and fungi (98). Most polysaccharides contain hydrophilic groups such as hydroxyl, carboxyl, and amino, which can form noncovalent bonds with intestinal mucus and promote the absorption of bioactive peptides (Figure 2C). As bioactive peptides contain amphipathic substances (that harbor both negatively and positively charged molecules) similar to proteins, they can also electrostatically interact with ionic polysaccharides with positive or negative charge to form associative complexes (99). Due to their effective biological activity, non-toxic, hydrophilic, good biocompatibility and biodegradability, and extensive diversity in structure and properties (100), polysaccharide-based nanomaterials are widely used in intracellular drug delivery as one of the most practical materials in nanomedicine field (Table 5).

**Table 5:** Application and characterization of polysaccharide nanomaterials.

**Delivery system**	**Utilization**	**Advantage**	**Disadvantage**	**Application**	**References**
Soluble soybean polysaccharide (SSPS)-based nanoparticles	To improve the long-lasting antimicrobial activity of Nisin (a food preservative)	1. High security 2. Good biocompatibility 3. Good biodegradability 4. Good pharmacokinetics and drug release characteristics	1. Most of the research for polysaccharide-based NPs is confined to the preclinical setting no commercial formulations of these systems are available 2. Poor bioavailability 3. Low target specificity 4. Limited stability	1. Polysaccharide NPs against chronic inflammatory disorders including rheumatoid arthritis (RA), diabetes and organ fibrosis 2. Delivery of anticancer agents,proteins/pepties, growth factors, antibiotics, anti-inflammatory and other drugs, as well as a strategy in both vaccine delivery and gene therapy	(98) (99) (100) (133) (134) (135)
n-trimethy chitosan (TMC) and sodium alginate (SA)	To maintain 41.76% of the bioactivity of peanut peptide (PP)				
Chitosan nanoparticles (CS NPs)	To adsorb the peptide as 70%				
Chitosan microparticles (CH MPs)	EE: 76% LC: 0.46%				

### Practical application of polysaccharides nanomaterial

The CS-based polysaccharide nanomaterial is prepared by the interaction between positively charged CS and negatively charged chlorinated polyethylene (PEC) through charges. It is considered to be a mild drug delivery method because no organic solvent or ultrasonic treatment is required (101). Yu et al. (102) developed a new type of CS-protamine nanoparticles for the anticancer effect of nuclear-targeted delivery of 5-fluorouracil (5-FU). Compared with free 5-FU, 5-FU-CS-protamine nanoparticles had the strongest cytotoxicity on tumor A549 cells and HeLa cells. In addition, an increase in apoptosis rate was also observed. It is worth noting that 5-FU-CS-protamine NPS can significantly reduce the tumor volume of BALB/c HeLa cell xenograft mice. Gao et al. (103) studied the combined delivery of irinotecan (IRN) and 5-FU by chitosan-hyaluronic acid (CS-HA) hybrid nanoparticles for the targeted treatment of gastric cancer. IRN/5-FU-CS-HA-NPs enhanced the killing effect on human gastric cancer cells *in vitro*, enhanced the antitumor activity *in vivo* of tumor-bearing mice, and had good antitumor activity. On the other hand, under extreme conditions, such as high temperature, the polysaccharide wall is susceptible to reacting with the peptide to form complex products (e.g., Maillard reaction products), which can be potentially toxic and also deplete the bioactive peptides. In order to circumvent this challenge, the reactive functional groups of polysaccharides have been modified such as carboxymethylation to produce relatively inert carriers. The molecular structure of polysaccharides contributes to their stability as carriers during the production and processing of encapsulated products (104, 105). Nasri et.al encapsulate goby fish protein hydrolysate (GPH), endowed with antioxidant activity, through an ionic gelation process using blue crab chitosan and tripolyphosphate anions and to evaluate the structural, thermal, and antioxidant properties of the elaborated microparticles. The increase in loaded GPH concentration led to an increase in encapsulation efficiency and a reduction in particle size. Furthermore, thermogravimetric analysis (TGA) profiles revealed the enhanced thermal stability of encapsulated biopeptides compared to the free ones (106).

Chitosan is a cationic polymer that allows electrostatic interaction with negatively charged sialic acid residues on the mucosal surface (107). Based on this principle, PLGA and FA-modified CS (FA-CS) composite nanocarriers were prepared by the electrostatic self-assembly method. The system can protect insulin against digestive enzymes and reduce the toxicity to HT-29 cells. *In vivo* experiments further showed that the system had an obvious hypoglycemic effect on diabetic mice, and the prepared composite nanocarriers were expected to become drug carriers for oral insulin and other biological macromolecules (108). Fish-purified antioxidant peptide (AOP)-loaded chitosan nanoparticles (CSNPs) were synthesized for Fourier transform infra-red (FT-IR) analysis, which demonstrated that the electrostatic interactions between biomolecule and polymer were considered as the driving force for peptide-loaded NPs formation (109). Similarly, sulfhydryl-modified low-molecular-weight chitosan was anchored to the lysine residue of insulin. These protein/chitosan conjugates have also proved to protect proteins against proteolytic enzymes, resulting in increased oral bioavailability of protein (110). PLGA covered with glycol-chitosan was used to encapsulate an HIV fusion inhibitor peptide (E2). Furthermore, the assessments *ex vivo* and *in vivo* were carried out to examine the ability of NPs to reach the vaginal epithelium. The penetration rate was measured in a swine model using confocal microscopy. E2-loaded NPs have interesting physicochemical and morphological features that provide them with mobility across the mucus, to reach the vaginal epithelium and release E2 (111). The W9-peptide, a tumor necrosis factor (TNF), was used to study the feasibility of cholesterol-bearing pullulan (CHP) nanogel as the drug delivery system. It found CHP nanogel could form a complex with the W9-peptide and prevents its aggregation *in vitro* (112). It suggested that the CHP nanogel worked as a suitable carrier for the W9 peptide. Santos et al. found that antimicrobial peptide (AMP) nisin has a wide range of action against pathogens and can treat diabetic foot ulcers (DFU) in patients with diabetes. However, AMP will be degraded or inactivated before reaching the target at the treatment concentration. Natural polysaccharide guar gum gel as the delivery system loading AMP can be used to treat systemic bacterial skin infections, especially those promoted by pathogens so that the sensitivity of bacteria to current antibiotics is reduced (113).

## Hydrogel delivery system

### Structural characterization of hydrogel delivery system

Hydrogel has three-dimensional networks of water swelling made of polymers, proteins, small molecules, or colloids (Figure 2D), and the obtained hydrogels show ultra-stable properties in highly acidic or alkaline aqueous solutions (114). The network of a hydrogel is established *via* covalent bonds or noncovalent interactions. Noncovalent interactions mainly include physical entanglements, hydrogen bonds, hydrophobic interactions, supramolecular interactions, electrostatic interactions, and coordination interactions (115). Hydrogel has adjustable physical properties. It prevents the degradation of unstable drugs as an effective drug delivery system. The versatility and diversity of hydrogels extend their applications to targeted drug delivery, wound dressings, contact lenses, and tissue engineering (Table 6). They contain 90% water and are highly porous to accommodate drugs for delivery and promote the controlled release (116).

**Table 6:** Application and characterization of hydrogels.

**Delivery system**	**Utilization**	**Advantage**	**Disadvantage**	**Application**	**References**
Hp-functionalized hydrogel system	More than 85% of Hp-bound basic fibroblast growth factor (bFGF) maintained bioactivity	1. Good biocompatibility 2. Stronger local drug penetration 3. Better drug bioavailability	1. Poor mechanical strength 2. Low specificity 3. Poor protein storage capacity	1. Anti-tumor drug delivery, transdermal drug delivery, ocular drug delivery, nasal drug delivery, and buccal drug delivery 2. Carrier for sustained release of peptide	(114) (115) (116) (136) (137) (138) (139) (140)
A hydrogel based on maleimide-bearing Hp and thiol-star-shaped polyethylene glycol (PEG) peptide conjugates crosslinked by a Michael-type addition	To keep >97% of the loaded vascular endothelial growth factor (VEGF)				
Hydrogel containing 3.3% aldehyde-modified PEG	Favorable insulin release (80% after 3 h)				

### Practical application of hydrogel delivery system

They developed a novel melittin-RADA_28_ (MR) hydrogel, composed of RADA_28_ and melittin, in order to promote the membrane permeability of tumor cells with the membrane-disrupting ability of melittin. It demonstrated that the formed melittin-RADA_28_-Lys-Leu-Ala-Lys-Leu-Ala-Lys-Lys-Leu-Ala-Lys-Leu-Ala-Lys (KLA) peptide (MRP) hydrogel has a nanofiber structure, sustained release, and attenuated hemolysis effects. Compared with free KLA, the MRP hydrogel markedly increased the cellular accumulation of KLA, produced the highest ratio of depolarized mitochondrial membrane, and decreased cell viability *in vitro* (117). The antibacterial effect of different concentrations of RADA16 on *Staphylococcus aureus, Candida albicans*, and *Escherichia coli* were compared. It was found that 0.25% of peptide-loaded RADA_16_ hydrogel could play a good antibacterial activity in a short time (118). The stable structure formed by RADA_16_ hydrogel provided an important carrier platform for the continuous release of antimicrobial peptides, which not only inhibited the reproduction of *S. aureus*, but also promoted the proliferation of bone marrow mesenchymal stem cells to a certain extent, and enhanced their osteogenic ability (119).

Dextran nanogels can be exploited as carriers for sustained/controlled release of bioactive molecules. Molinos et al. prepared a new bidimensional composite hydrogel, made of oxidized dextrin incorporating dextrin nanogel (oDex nanogel). It provides continuous delivery of protein and prevents problems of cyclic variations in the protein concentration in the blood with time. In addition, it offers maximum pharmacological efficiency at a minimum drug dose, reducing administration frequency and improving patient compliance with the therapy (120). Hirakura et al. reported a novel injectable hybrid hyaluronan (HA) hydrogel system of physically encapsulated CHP nanogels for sustained delivery of protein. Furthermore, a new hybrid hydrogel composed of CHP nanogels presented chaperone-like protection for fragile protein drugs and chemically cross-linked HA as the biocompatible controlled release matrix. CHP/protein complex nanogels were released from the hybrid hydrogels in a sustained manner *in vitro* and *in vivo* (121). The main challenge is to design a hydrogel system capable of providing drug release for several months and complete clearance at the end of drug release or in a relatively short period of time to avoid matrix accumulation (122).

## Concluding remarks and future perspectives

The application of nanotechnology in biomedicine and functional food to improve human health is indispensable. The properties of nanocarriers with different materials can meet the different needs of bioactive peptide delivery. It shows great advantages in solving targeting, stability, loading, release rate, biodegradability, and biocompatibility of bioactive peptides. Certain problems to be solved urgently in the nanotechnology of bioactive peptides are as follows: (1) Due to the unique structure, it is difficult to separate and purify peptide drugs, and the effects of various modification methods and solvents may change the molecular structure of bioactive peptides, resulting in the loss of biological functions. (2) Although many nanocarriers have been shown to be low-toxic or non-toxic *in vitro*, it is still unclear whether they will affect organisms or even humans, and the preclinical acute and chronic toxicity research must be paid enough attention by researchers. (3) It is worth pondering whether bioactive peptides can effectively cross multiple biological barriers *in vivo* due to their complex forms of polymorphism and conformation. (4) After most bioactive peptides enter cells, they need to undergo intracellular transport and intracellular release to exert physiological and pharmacological effects depending on delivery efficiency. (5) It is another important problem that how nanocarriers adapt to individual differences. In summary, in the future, efforts should be made to further verify the physiological conditions such as pH value, protease level, mucus, epithelial barrier, and intestinal transport pathway, which can guide the formulation optimization and design of new delivery systems to improve stability and bioavailability of bioactive peptides after oral administration.

## Author contributions

XZ composed the outline and drafted the manuscript. QW reviewed and approved the submitted version. XL, YZ, QZ, and YY read and approved the final version. All authors had full access to data and revised and approved the manuscript for publication.

## Funding

We gratefully acknowledge the financial support from the National Natural Scientific Foundation of China (Grant Number 32202573) and the Natural Science Foundation of Hunan Province (Grant Number 2022JJ50028).

## Conflict of interest

The authors declare that the research was conducted in the absence of any commercial or financial relationships that could be construed as a potential conflict of interest.

## Publisher's note

All claims expressed in this article are solely those of the authors and do not necessarily represent those of their affiliated organizations, or those of the publisher, the editors and the reviewers. Any product that may be evaluated in this article, or claim that may be made by its manufacturer, is not guaranteed or endorsed by the publisher.
